# Some Points on the Reduction of Hernia

**Published:** 1883-02-20

**Authors:** J. S. Wight


### SOME POINTS ON THE REDUCTION OF HERNIA.

By J. S. Wight, M. D.

Seeing that the major operation, or opening the sac, in a case of hernia is one that may involve great danger, and seeing that the minor operation, in which the sac is not opened, may involve some danger, and seeing that the *taxis* is a safe procedure, especially when it is successful, any expedients that will enable the surgeon to reduce a greater number of hernia, so that fewer operations will be required, will be the means of saving life. In this statement it is implied that cases of hernia are operated on that do not require an operation, and it must be admitted that it is not good practice to operate on a hernia that can be reduced by taxis.

The method of taxis for reducing a hernia—especially one that is strangulated—that I have adopted and advise, may be described as follows:

1. As far as possible, grasp the hernial tumor with one hand: this can generally be readily done, except when the tumor is very large. The right hand will be best adapted for this purpose.

2. Now take hold of the neck of the hernial sac with the thumb and fingers of the left hand, in close proximity to the ring of constricting tissues, which can generally be readily distinguished.

3. Then make gentle traction on the hernial tumor by means of the right hand. when two effects will generally supervene: (1) The hernia will be drawn out a little and liberated from the ring of constricting tissues, and (2) some of the fluid contents, and may be some of the solid contents, of the sac may be felt going through the hernial canal into the abdominal cavity. As the hand pulls on the tumor, it will compress it at the same time, and thus tend to express the contents of the sac; and the contents of the sac will be more apt to be expressed because the hernia is liberated from its constriction.

4. The thumb and fingers of the left hand, as it were, supplement the hernial canal. as they are near the constricting tissues, so that the sac and its contents will be prevented from expanding just outside of the outer end of the hernial canal. In one instance the thumb and fingers will accurately guide the hernial contents into the hernial canal, and in the other instance the hernial contents will swell out around the outer end of the hernial canal. In the latter instance the reduction of the dislocated intestine will be obstructed, and in the former instance its reduction will be greatly facilitated.

5. When the fluid contents of the sac begin to *go back*, then the solid contents of the sac will also begin to *go back*. The left hand of the surgeon must still continue the work it has begun, but the right hand must now. in addition to firmly grasping the hernial tumor, begin to push this tumor toward the external ring, in between the grasp of the thumb and fingers of the left hand, when, generally, little by little, and sometimes suddenly, the dislocated intestine will be reduced. Of course, the rules of position and relaxation in regard to the patient should be put in force. When this method of taxis is properly carried out, it will, no doubt, diminish the number of operations for strangulated hernia.

6. In this place I may draw attention to this method of taxis, for the purpose of reducing a hernia when the minor operation is performed, since the constriction may be outside the neck of the sac. Also, I may call attention to the fact that I have sometimes expanded and stretched, or, perhaps, torn more or less, the constricting band of tissues about the neck of the sac, by means of my finger, which has been pushed up under the edge of this band, carrying the tegumentary tissues before it, thus enabling me to reduce a hernia because the canal has been enlarged. At times I have found this a most valuable expedient, and have never known it do any harm.

In order to illustrate the procedure above described, two cases of femoral hernia may be related:

I. Mrs. S., widow, 63 years of age, was seen by me for the first time January 6, 1882. She had a strangulated femoral hernia on the left side, about as large as one's fist. The swelling had been down about a week; the patient had been vomiting for three or four days; the temperature was about 90°; the pulse was about 100; the abdomen was soft and was not tender; the bowels had not moved for a week; there was no special tenderness about the tumor; the patient was anxious and depressed in spirits. Two days before I saw this patient a physician visited her, and did not succeed in reducing the hernia: another physician saw the patient on the evening before I saw her, and did not succeed in reducing the hernia. The patient was under the impression that an operation must be performed, and that she would die.

I saw this patient about five o'clock, p. m., January 6, 1882, and put in practice the method of taxis above described, and reduced the hernia in about fifteen minutes; ordered rest, anodynes, and a mild diet, followed in two days by a dose of castor oil. This patient made a rapid and excellent recovery.

II. Mrs. M., widow, 42 years of age, much addicted to alcoholic drink, was seen first by me January 16, 1882. She had a strangulated femoral hernia on the left side nearly as large as one's fist; it had been down for nearly one week; the patient had been vomiting for two days; her temperature was 98½°; the pulse was about 110; the abdomen was soft, and the furrow in the inguinal region was deep, as there was much adipose tissue. Her physician advised an operation, and was of the opinion that she would die without an operation. The patient declined an operation.

I saw the patient about half-past one, p. m., of January 16th, and in about ten minutes reduced the hernia by means of the method of taxis above described. In the evening the patient's bowels moved three times. Rest and a mild diet completed the cure in a few days.—*Soc. Co. Kings.*

---

**Anæsthesia of the Pharynx.**—M. Du Cazal remarks that tincture of coca is an excellent medicament to cause anæsthesia of the pharynx. This can be secured by simply painting the mucous membrane. This fact is of interest to all who use the laryngoscope.

